# RETRACTED: Durairaju et al. Synthesis and Characterization of Pyridine-Grafted Copolymers of Acrylic Acid–Styrene Derivatives for Antimicrobial and Fluorescence Applications. *Micromachines* 2021, *12*, 672

**DOI:** 10.3390/mi16111220

**Published:** 2025-10-27

**Authors:** Periyan Durairaju, Chinnasamy Umarani, Jothi Ramalingam Rajabather, Amer M. Alanazi, Govindasami Periyasami, Lee D. Wilson

**Affiliations:** 1 Department of Chemistry, Thiruvalluvar Government Arts College, Rasipuram 636007, Tamilnadu, India; 2 Department of Chemistry, Government Arts College (Autonomous), Salem 636007, Tamilnadu, India; sachuutay@gmail.com; 3 Chemistry Department, College of Sciences, King Saud University, Riyadh 11451, Saudi Arabia; jrajabathar@ksu.edu.sa (J.R.R.); pkandhan@ksu.edu.sa (G.P.); 4 Pharmaceutical Chemistry Department, College of Pharmacy, King Saud University, Riyadh 11451, Saudi Arabia; amalanazi@ksu.edu.sa; 5 Department of Chemistry, University of Saskatchewan, 110 Science Place—Room 165 Thorvaldson Bldg., Saskatoon, SK S7N 5C9, Canada

The journal retracts the article “Synthesis and Characterization of Pyridine-Grafted Copolymers of Acrylic Acid–Styrene Derivatives for Antimicrobial and Fluorescence Applications” [1] cited above.

Following publication, concerns were brought to the attention of the Editorial Office regarding image anomalies contained within this publication [1].

Adhering to our standard procedure, an investigation was conducted by the Editorial Office and Editorial Board that identified indications of inappropriate editing and duplication between figures presented in this article [1] and an earlier publication [2], produced by a similar research group and presenting different experimental conditions.

While the authors collaborated throughout the process, they were unable to satisfactorily address the above-mentioned concerns or submit raw materials that the Editorial Board deemed sufficient to verify the integrity of the images. Consequently, the Editorial Board has lost confidence in the reliability of the findings and has decided to retract this publication, as per MDPI’s retraction policy (https://www.mdpi.com/ethics#_bookmark30).

This retraction was approved by the Section Editor-in-Chief of the journal *Micromachines*.

The authors did not agree to this retraction.

## References

[1] Durairaju P., Umarani C., Rajabather J.R., Alanazi A.M., Periyasami G., Wilson L.D. (2021). RETRACTED: Synthesis and Characterization of Pyridine-Grafted Copolymers of Acrylic Acid–Styrene Derivatives for Antimicrobial and Fluorescence Applications. Micromachines.

[2] Ramalingam R.J., Vaali-Mohammed M.-A., Al-Lohedan H.A., Appaturi J.N. (2017). Synthesis and bio-physical characterization of Silver nanoparticle and Ag-mesoporous MnO_2_ nanocomposite for anti-microbial and anti-cancer activity. J. Mol. Liq..

